# Supplementary material for: Effects of cutaneous leishmaniasis on health-related quality of life: A longitudinal approach
Source: PLoS One. 2025 Oct 8;20(10):e0334215. doi: 10.1371/journal.pone.0334215 (PMC12507214; doi:10.1371/journal.pone.0334215)
Supplement: Attachment — Submitted filename: I think this is a well written article.docx [file pone.0334215.s001.docx]

I think this is a well written article. However I have a few comments which needs to be addressed.

1. How were the patients diagnosed? Which according to them was the most satisfying and accurate?

2.Which according to the patients was the safest and best drug for treatment as far as satisfaction and permanent cure are concerned?

4.What was the definition of complete cure in these patients?

5. Please define the HIV status and other clinical parameters of these patients.

6. For how long were these patients hospitalised?

7. Were these patients followed up, if so for how long?

8. The english needs to be improved.
