# Supplementary material for: Effects of cutaneous leishmaniasis on health-related quality of life: A longitudinal approach
Source: PLoS One. 2025 Oct 8;20(10):e0334215. doi: 10.1371/journal.pone.0334215 (PMC12507214; doi:10.1371/journal.pone.0334215)
Supplement: Attachment — Submitted filename: Response to Reviewers_18.09.25.docx [file pone.0334215.s003.docx]

September 18, 2025

Dear Editor,

PLOS ONE

We would like to thank the reviewers for their careful review of the manuscript *"Effects of cutaneous leishmaniasis on the health-related quality of life: a longitudinal approach*" (PONE-D-25-35705), this opportunity to improve our work makes it even more robust and useful to the scientific community - our sole goal.

We did our best to improve the text accordingly. The modifications implemented in this revised version are summarized below together with specific answers to the questions placed by the reviewers. We reinforced that the funders had no role in study design, data collection and analysis, decision to publish, or preparation of the manuscript.

As guidance for the editorial process: the changes highlighted in blue within the revised manuscript correspond to the alterations made in response to the reviewers’ comments, while the changes highlighted in red correspond to the professional English language revision.

We hope the reviewers find our responses to their comments satisfactory. Please let us know if any further improvements to the manuscript are needed.

Sincerely yours,

Corresponding author:

Sarah Nascimento Silva

Instituto René Rachou - Fundação Oswaldo Cruz – Fiocruz Minas

Avenida Augusto de Lima, 1715

30190-001, Belo Horizonte, MG, Brazil

Phone/fax:	+ 55 31 3349-7782 - E-mail: [sarah.nascimento@fiocruz.br](mailto:sarah.nascimento@fiocruz.br)

**Answer Letter to Reviewers**

| **Local** | **Reviewer comment** | **Author response** | **Alterations in manuscript** |
| --- | --- | --- | --- |
| **Reviewer #1** | | | |
| Overall impression | 1. How were the patients diagnosed? Which according to them was the most satisfying and accurate?  2.Which according to the patients was the safest and best drug for treatment as far as satisfaction and permanent cure are concerned?  3.What was the definition of complete cure in these patients?  4. Please define the HIV status and other clinical parameters of these patients.  5. For how long were these patients hospitalised?  6. Were these patients followed up, if so for how long?  7. The english needs to be improved. | We sincerely thank the reviewer for these thoughtful and detailed comments, which we carefully considered. We provide clarifications below and describe the minor modifications made in the manuscript:    Diagnosis: As described in the Methods section (line 118), all participants were included after parasitological confirmation of cutaneous leishmaniasis, following the standard protocol of the Brazilian Ministry of Health. No changes to the manuscript were required.    Patient perception of treatment: Although patients were asked about their impressions regarding treatment, this study did not aim to compare satisfaction across different drugs. Treatment selection was guided by clinical manifestations, comorbidities, and medical judgment. Patient perspectives were captured more broadly through the validated CLIQ instrument, which assesses multiple dimensions of quality of life. A clarifying statement has been added in the Methods to reinforce this point (line 159).    Definition of cure: According to Brazilian Ministry of Health guidelines, cure is defined as complete epithelialization of all lesions and disappearance of crusts, scaling, infiltration, and erythema. However, cure was not used as a study variable or endpoint in this research, as it was not within the scope of our research. Instead, our analyses focused on longitudinal changes in health-related quality of life (HRQoL), regardless of clinical outcome.    HIV and clinical parameters: We acknowledge the importance of HIV status and other clinical parameters in the management of leishmaniasis. At the reference center where the study was conducted, all patients routinely undergo serological testing for HIV, hepatitis, and syphilis, as well as baseline laboratory evaluations (renal, hepatic, and hematologic parameters) prior to treatment initiation. However, these data were not included in the present study, as they were not within the primary scope of our analyses, which focused on longitudinal changes in HRQoL. We agree that future studies specifically designed to explore the impact of HIV status and other comorbidities on HRQoL in this population would be valuable.    Hospitalization: We appreciate the reviewer’s observation. Hospitalization was required only for patients treated with amphotericin B, with an average stay of 10–12 days depending on clinical response. All other therapies were administered on an outpatient basis, either at the reference center or within the public health network. To address this point, a clarifying paragraph has been added to the Discussion section (line 334).  Follow-up: Patients were prospectively followed from study inclusion until 180 days, as specified in the Methods section (line 127 to 133).    Language revision: The manuscript has undergone professional proofreading by a specialized scientific translation company to improve clarity and sentence flow. | Clarifications were incorporated into the Methods and Discussion sections to address the reviewer’s comments, and these modifications are highlighted in red in the revised manuscript:  (Lines 121-123; lines 136-137; lines 159-162; lines 366-374).  All text was passed to a professional English-language editor for revision. |
| **Reviewer #2** | | | |
| Overall impression | This is a well-designed and clearly reported longitudinal study that fills an important gap in understanding the health-related quality of life (HRQoL) of cutaneous leishmaniasis patients. The results are valuable for informing patient-centered care and guiding public health interventions. | We appreciate the opportunity to receive this feedback and have a chance to improve our manuscript. We would like to thank the reviewer for their efforts and their comments. | - |
| Abstract | Please include an introductory section to provide background on the study area and the rationale for the research. | Thank you for this suggestion. We have added an introductory section in the abstract to provide the necessary background and rationale for the study. | Abstract, line 37 to 42. |
| Terminology | Ensure that Health-Related Quality of Life (HRQoL) is written in full before introducing the abbreviation in the abstract. | We appreciate this observation. The term Health-Related Quality of Life (HRQoL) is now written in full before the abbreviation in the Abstract Introduction, as suggested. | Abstract Introduction, line 39. |
| Ethical Considerations | The study population includes participants aged 12 years and above. Are 12-year-olds eligible to provide informed consent independently in Brazil? According to international ethical guidance (e.g., Declaration of Helsinki; CIOMS, 2016), children and adolescents should provide assent, while parental or guardian permission is required. Please clarify whether parental consent and child assent were obtained. | We thank the reviewer for this important observation. We have clarified this point in the Methods section. | The revised text in the Methods Section (line 107) now states: “For individuals aged 12 to 17 years, both parental or guardian consent and adolescent assent were obtained, in accordance with Brazilian regulations and international ethical standards (Declaration of Helsinki, 2013; CIOMS, 2016).” |
| Methodology Consistency | In the results, it states: “A total of 143 patients diagnosed with cutaneous, mucocutaneous (MCL), or mucosal leishmaniasis.” Please ensure that the different clinical forms of leishmaniasis are consistently described in the methodology as well. | We thank the reviewer for this helpful observation. | We have revised the Methods section (Line 135) to ensure consistency in terminology. The text now specifies: *“Sociodemographic information (sex, age, education) and clinical data—including clinical form of leishmaniasis [cutaneous, mucocutaneous (MCL), or mucosal leishmaniasis (ML)], presence of comorbidities, number of lesions, presence of ulceration or bacterial infection, case type (new or relapse) treatment used, and occurrence of adverse events—were recorded by the healthcare professional responsible for treatment and extracted from medical records using a structured form.”* |
| Proofreading | The manuscript would benefit from careful proofreading to improve sentence flow and clarity. | We appreciate this observation. The manuscript has now been professionally revised by a specialized company to improve sentence flow and clarity. | The entire manuscript has been carefully proofread and revised by a professional company specialized in scientific translation. |
| Sample Size | Please explain how the sample size was determined. Was a calculation performed or was it based on available cases? | We appreciate this comment. No formal sample size calculation was performed. The study included all patients with confirmed cutaneous leishmaniasis who were available and agreed to participate at the reference center between October 2020 and April 2023. | This clarification about sample size calculation has been added to the Methods section (line 121). |
| Inclusion criteria | Methodology-The inclusion criteria in the Methods section states that participants aged 12 years and older were eligible. However, in the Results section (Table 1), the youngest age category is reported as 15–29 years. This discrepancy needs clarification.  a) Were any participants aged 12–14 recruited?  b)If not, please explain why this group was included in the eligibility criteria but absent from the results.  c)If they were excluded post hoc, provide justification (e.g., ethical reasons, consent/assent procedures, or no eligible participants in this age band). | We appreciate the reviewer’s careful observation. Although the inclusion criteria allowed participants aged 12 years and older, no individuals aged 12–14 years presented to the reference center or met the study conditions during the recruitment period. Therefore, the youngest participants enrolled were 15 years old. This explains why the first age category presented in the Results and Table 1 is 15–29 years. No participants were excluded post hoc based on age. | A clarifying statement was added to the Results section (Line 183) to specify that, although the eligibility criteria included participants from 12 years old, no patients aged 12–14 years were recruited during the study period. |
| Loss to Follow-up | The reported 16% loss to follow-up is acceptable; however, presenting a brief comparison of baseline characteristics between participants who completed the study and those lost to follow-up would strengthen confidence in the findings. Were there notable demographic or clinical differences? The manuscript reports 143 participants in the socio-demographics section, despite a 16% loss to follow-up (final n=120). Please clarify this discrepancy. | We thank the reviewer for this valuable observation and acknowledge the oversight. To address this, Table 1 has been revised to present the baseline sociodemographic and clinical characteristics of participants who completed follow-up (n=120). | Table 1 has been revised to present the baseline sociodemographic and clinical characteristics of participants who completed follow-up (n=120). |
| Discussion Citations | Several statements in the discussion would benefit from citations, particularly the finding that financial burden and social isolation persist despite treatment. Strengthening these points with references and linking them to policy implications would improve the discussion.  Discussion Line 287–288 – Please provide appropriate citations to support this statement. | We thank the reviewer for this valuable comment. The Discussion section has been strengthened with the inclusion of PubMed-indexed references that corroborate the persistence of financial burden, social isolation, and heterogeneous patient experiences despite treatment. | Additional references have been inserted into the Discussion section (References 6, 7, 19 and 22). |
